# Study on the Mechanism of Hydrolyzed Seawater Pearl Tablet in Treating Chronic Sleep Deprivation Mice Model

**DOI:** 10.2174/1871530323666230206160722

**Published:** 2023-05-17

**Authors:** Fei Luo, Siyin Han, Meng Xia, Zhenxing Chen, Peng Liu, Jiang Lin

**Affiliations:** 1 School of Basic Medical Science, Guangxi University of Chinese Medicine, Nanning 530200, China

**Keywords:** Hydrolyzed seawater pearl tablet, chronic sleep deprivation, hippocsampus, oxidative stress, cell apoptosis, microbiota

## Abstract

**Background:**

Modern lifestyle increasingly deprives people from sleep to different degrees. Long-term sleep deprivation will facilitate body’s pathological behaviors, such as lethargy, depression, and anorexia.

**Objective:**

This study is an investigation into the mechanism of hydrolyzed seawater pearl tablet in treating chronic sleep deprivation mice model.

**Methods:**

The chronic sleep deprivation model was established involving C57BL/6mice; the body weight, behavioral characteristics, hippocampal structure, oxidative stress, apoptosis-related protein expression, and intestinal bacteria in mice were assessed to characterise hydrolyzed seawater pearl tablet.

**Results:**

Hydrolyzed seawater pearl tablet significantly accelerated body weight, open field test score, and sugar water preference rate (*P* < 0.05), alleviated the structural damage of hippocampus, reduced the content of MDA (*P* < 0.05), Bax protein expression, increased the content of GSH (*P* < 0.05), the activities of SOD, GSH-Px, and Bcl-2 protein expression in the hippocampus, increased the *Escherichia coli*, Bacteroides, Bifidobacterium and Lactobacillus (*P* < 0.05), which are beneficial bacteria in the intestine, in chronic sleep deprivation mice, and reduced the amount of Clostridium perfringens (*P* < 0.05), which are harmful bacteria in the intestine.

**Conclusion:**

Hydrolyzed seawater pearl tablet can improve the depression-like mental state of mice caused by chronic sleep deprivation. The mechanism involves improving the antioxidant activity of the hippocampus to eliminate the excessive ROS, which inhibits cell apoptosis and alleviates tissue structure damage. Meanwhile, it may also be involved in adjusting the microbiota level and improving the mental and behavioral activities of chronic sleep deprivation mice through the intestine-brain axis.

## 1 INTRODUCTION

Sleep is the basic element to maintain the normal physiological and psychological activities of the body. Modern lifestyle increasingly deprives people from sleep to different degrees. As a strong stress stimulus, long-term sleep deprivation will affect the body's behavior and physiological function, increase systemic diseases, lead to chronic fatigue syndrome and cognitive decline [1], and facilitate body’s pathological behaviors, such as lethargy, depression, and anorexia. Some studies have shown that sleep deprivation can induce oxidative stress, which is mainly manifested in that the body is in a high metabolic state after sleep deprivation, and increased energy consumption leads to excessive production of free radicals (ROS) [2, 3]. At the same time, it reduces the capacity of the body's antioxidant defense system, leading to a decrease in the activity of antioxidant enzymes and the content of antioxidant complexes [4-5]. Oxidative stress is the imbalance between metabolic oxidation and antioxidants in the body, which can cause oxidative damage to DNA, lipids, and proteins, and then cause apoptosis and tissue damage [6]. In addition, the brain is closely connected with the intestine through 200-600 million neurons, forming an intestine-brain axis [7], and the two interact. Under stress, the microbiota is disordered, and the structure and composition of specific flora will change [8], thus affecting the brain and behavior [9]. The invasion of pathogenic bacteria will increase anxiety-like and depression-like behaviors, while beneficial bacteria will alleviate the symptoms of such mental diseases [10]. It is speculated that drugs may improve the mental disease-like behavior caused by chronic sleep deprivation through antioxidant stress and regulating microbiota.

The ancient Pharmacopoeia of the People's Republic of China and the Dictionary of Traditional Chinese Medicine both recorded that “pearl is effective in detoxification and muscle regeneration, calming nerves, brightening eyes, and eliminating pannus formation”. In recent years, Han *et al*. have found that pearl powder can prevent and treat skin photoaging in mice, and its mechanism involves the removal of excessive ROS from photoaging skin [11, 12]. Xia *et al*. have demonstrated pearl powder to reduce stress response by correcting aberrant gene expression in sleep deprivation model rats [13]. As a matter of fact, pearls are difficult to dissolve in water. When taken orally or externally, the effective ingredients that can be absorbed by the human body are very limited. After applying the low-temperature hydrolysis technology, the hydrolyzed pearl not only preserves the whole ingredients of pearls, but also enables the active and effective ingredients to be absorbed by the human body to the maximum extent, giving full play to the physiological effects of pearls, which is suitable for clinical application.

In the early stage, our research group selected seawater pearls for hydrolysis and obtained a series of products, such as seawater pearl hydrolysate and hydrolyzed seawater pearl tablet, and successfully developed the hydrolyzed pearl technology. It has been found that seawater pearl hydrolysate can remove excessive ROS from photoaging cell induced by UVA [14a]. Hydrolyzed seawater pearl tablet is derived from seawater pearl hydrolyzed by enzymatic proteases following our patented technology. Hydrolyzed seawater pearl tablet is derived from seawater pearl hydrolyzed by enzymatic proteases following our patented technology [14b]. To date, it is being aimed to explain the clinical practices, find the bioactive property, and provide insight into the mode of action. Therefore, we studied the therapeutic mechanism of hydrolyzed seawater pearl tablet on chronic sleep deprivation model mice at the cellular level to detect oxidative stress, apoptosis-regulated proteins, and specific microbiota before and after hydrolyzed seawater pearl tablet intervention in chronic sleep deprivation model of mice.

## 2 MATERIALS AND METHODS

### 2.1 Experimental Animals and Medicine

Forty SPF grade C57BL/6 male mice with a body mass of 22±3 g were used in the experiment. They were purchased from Hunan Slake Jingda Experimental Animal Co., Ltd. (Changsha, China; permit number: SCXK (Xiang) 2019-0004). All experimental procedures were performed in accordance with the National Institutes of Health Guide for the Care and Use of Laboratory. The animals were kept in the clean animal laboratory with a temperature of 23 ± 1°C and a relative humidity of 55 ± 5% with a 12 h light/dark cycle. Prior to the study experiments, animals were provided adaptive feeding for 7 days. Hydrolyzed seawater pearl tablets (production approval number: 450522020014, product number: 20180102) were kindly donated by Baozhulin Ocean Technology Co., Ltd. (Beihai, China) and used throughout the study; Chen *et al*. analyzed the presence of amino acid in hydrolyzed seawater pearl tablet [15]. Estazolam tablets (product batch No.: 20200602) were purchased from Huazhong Pharmaceutical Co., Ltd. (Xiangyang, China).

### 2.2 Main Instruments and Chemicals

The sleep deprivation device is a simple sleep deprivation box modified from a mouse cage. Six small round-shaped platforms with a diameter of about 2.7 cm and a height of 6 cm are fixed at the bottom of the mouse cage. The platforms are made of PP plastic. Clean water is injected into the sleep deprivation box about 0.5 cm away from the top of the platform. The water temperature in the sleep deprivation box is kept at 26ed platforms with a diameter of about 2.7 cm and a height of 6 cm, and the water in the box is replaced regularly every day. The open field test box was purchased from Shanghai Biowill Co., Ltd. (Shanghai, China).

The following mentioned chemicals were used. Determination kits for BCA protein concentration, malondialdehyde (MDA), glutathione (GSH), superoxide dismutase (SOD), and glutathione peroxidase (GSH-Px) were purchased from Nanjing Jiancheng Bioengineering Institute (Nanjing, China); monoclonal antibodies, namely, anti-GAPDH, anti-Bcl-2, and anti-Bax, were purchased from Abcam (Milton, Cambridge, UK). Goat anti-rabbit IgG-horseradish peroxidase (HRP) secondary antibody was purchased from Aspen (Linden, Utah, USA). DNA extraction kit for fecal samples was obtained from Shanghai Solebar Co., Ltd, Shanghai, China. QRT-PCR reaction premix was received from ABI, San Diego, California, USA.

### 2.3 Grouping and Chronic Sleep Deprivation Treatment in Mice

Forty C57BL/6 male mice were fed adaptively for one week and randomly divided into four groups, namely, the normal (N) group, model (M) group, estazolam (ES) group, and hydrolyzed seawater pearl tablet (HSPT) group. Ten mice in each group were fed in the same cage. The N group was fed routinely without any intervention, and the other groups were treated with chronic sleep deprivation. Chronic sleep deprivation mice were subjected to chronic sleep deprivation stress for 7 days by using a sleep deprivation device, and then a chronic sleep deprivation mouse model was established. The details are as follows: mice were deprived of 20 hours’ sleep every day, followed by 4 hours of rest recovery time. During the rest period, they ate and drank normally, and the sleep deprivation duration was 7 days in total.

### 2.4 Administration Treatment in Mice and Sampling

After establishing the chronic sleep deprivation mouse model, each group was given different drugs by gavage for 14 days. According to the dose conversion formula between mice and adults (70kg), the daily dose of mice was obtained. The dose for the HSPT group was 0.16g/kg hydrolyzed seawater pearl tablet, and the dose for the ES group was 0.26mg/kg estazolam. 0.5ml of normal saline was added for gavage treatment for both groups. The model group and the normal group were gavaged with an equal volume of normal saline.

After the experiment, mice were weighed, and behavioral tests were performed. Samples were taken the next day after the experiment. The mice were killed, the hippocampal tissue of the brain was taken out, and part of it was stored at -80°C to detect *oxidative stress and apoptosis-regulated protein expression*; other parts were fixed in 4% paraformaldehyde for making histopathological tissue sections. Colonic feces were collected and stored at -80°C to detect specific microbiota.

### 2.5 Open Field Experiment

After the experiment, the mice were placed in the center of the open field test box, video recording was started, and the behavior and activities of the mice in the open field within 5 minutes were observed. The observation and recording items included the number of small blocks they went through on the ground when the mice were active, and the entry made through more than 3 claws was taken as the standard and calculated as the horizontal movement score. The number of times the mice's two forefeet left the floor was taken as the score of vertical movement (no matter how long the mouse stands, until it puts down its two forefeet, it can be recorded as 1 point, and the score is calculated according to the number of times). This whole test process was conducted in a quiet environment away from light.

### 2.6 Sugar Water Preference Experiment

Sugar water preference test was conducted after the experiment. The specific process is as follows: first, the mice are trained to adapt to sugary drinking water. For the first 24 hours, two small bottles are placed in each cage at the same time, each containing 1% sucrose water. For the second 24 hours, one bottle of 1% sucrose water and the other bottle of pure water are exchanged every 12 hours. After the training, the mice are made to fast for 24 hours. Then, the sugar water preference experiment is carried out, and each cage of mice is given two bottles of water that have been weighed: one bottle of 1% sucrose water and the other bottle of pure water. After 15h (changing the bottle position at 8h halfway), the two bottles are taken away and weighed, the sugar water consumption and pure water consumption are calculated, and then the sugar water preference rate of mice is determined.

Sugar water preference rate = sugar water consumption/(sugar water consumption + pure water consumption).

### 2.7 Evaluation of Hippocampal Structure

Fixed hippocampal tissue sections were dehydrated in an ascending alcohol series (50% to 95% concentration). Dehydrated tissues were embedded in paraffin, sectioned into 5 *μ*m thick sections, and stained with hematoxylin and eosin (HE). Sections were then examined with a light microscope.

### 2.8 Detection of the Level of Oxidative Stress in Hippocampal Tissue

Hippocampal tissues, stored at -80°C, were homogenized with cooled Tris-HCl buffer at 1:5 (w/v), centrifuged at 4°C at 3,500 r/min for 10 min, and the supernatant was taken out for sample analysis. The activities of SOD and GSH-Px, and the contents of GSH and MDA in the hippocampal tissue were detected according to the operating instructions of the respective kits. The values have been expressed in relative units per mg of soluble protein.

### 2.9 Detection of Expression of *Apoptosis-regulated Proteins* in Hippocampal Tissue

Hippocampal tissues, stored at - 80°C, were homogenized with cooled protein extraction reagent at 1:10 (w/v).The homogenate was transferred to a centrifuge tube, vibrated and kept in an ice bath for 30 min, and then centrifuged at 4°C at 12,000 r/min for 5 min. The supernatant was collected, to which was added 5 × protein loading buffer of appropriate equivalent, and then immersed in boiling water at 100°C for 5 min as a protein sample. The separation gel and concentrated gel were prepared, and then the protein samples were added to the sampling wells. Transfer membrane filter paper and methanol-activated PVDF membrane were prepared, and the current was allowed to flow through the membrane at a constant rate of 300 mA. The transferred membrane was added to the sealing solution and sealed for 1 hour at room temperature. The blocking solution was removed, and the monoclonal antibodies, namely, anti-GAPDH, anti-Bcl-2, and anti-Bax, were added after dilution with a diluent for monoclonal antibodies and incubated at 4°C overnight. The diluted monoclonal antibodies were recovered and washed with TBST thrice for 5 min each time. The diluted goat anti-rabbit IgG-HRP secondary antibody was added and incubated at room temperature for 30 min. TBST was used for washing four times on a shaking table at room temperature for 5 min each time. Fresh mixed ECL solution was added to the protein side of the membrane, which was then exposed in a dark room. The film was archived, analyzed, and scanned.

### 2.10 DNA Expression Detection of five Kinds of Intestinal Bacteria using qRT-PCR

#### 2.10.1 DNA Extraction

0.2g of fecal sample in a 2 ml round-bottom centrifuge tube was weighed, and DNA was extracted according to the instructions of the fecal DNA extraction kit. The concentration and purity were determined by micro spectrophotometer: after zeroing the buffer eluent in the kit, the absorbance (*A*260) value was detected at 260 nm to determine the concentration. The absorbance (*A*280) value at 280 nm was detected, and the *A*260/*A*280 value was found to be between 1.7 and 1.9, indicating the DNA purity as qualified. The obtained fecal DNA was diluted to 40mg/L with DEPC water and stored at -80°C for future use.

#### 2.10.2 PCR Primer Design

According to the 16S rRNA gene sequences of the five kinds of intestinal bacteria investigated, the PCR primers of the corresponding bacterial genus were designed with primer design software Primer Premier 5.0, and the corresponding bacterial genus specificity of the primer sequences was compared in the BLAST gene bank (www.ncbi.nlm.nih.gov/ BLAST). Table **1** lists the primer sequences.

#### 2.10.3 qRT-PCR Response

20μL of reaction solution containing 10 μL SYBR Green premix, 0.3 μMol/l primer and 40 ng of template DNA was added to each well of the octuple tube, and finally supplemented with DEPC water. Each sample was set with one complex well. After the sample was added, the reaction tube was covered, mixed gently, and centrifuged briefly to ensure that all components remain at the bottom of the tube. qRT-PCR response was determined as follows: pre-denaturation at 95°C for 15 min, denaturation at 95°C for 10 s, annealing at 60°C for 30 s, and 72°C extension for 30 s, with a total of 40 cycles. The last cycle was 72°C for 2 min, and the melting curve was analyzed at 60~95°C. The amplification curve and dissolution curve of each bacterium are shown in Fig. (**1**) (A-E1, 2). The cycle threshold *C*t was detected, and analysis was conducted using statistical data expressed as 2^−ΔΔ^*^C^*^t^.

### 2.11 Statistical Analysis

Datas were all expressed as mean±standard error (SE). SPSS (version 17.0) (IBM, Armonk, NY, USA) was used to conduct statistical analysis. One-way ANOVA and Least Significant Difference (LSD) tests were used to conduct variation analysis among different groups. The difference was considered significant statistically when value with P<0.05 was obtained. All histograms were derived from Origin 8.6 software (OriginLab, Northampton, MA, USA).

## 3 RESULTS

### 3.1 Effect of Hydrolyzed Seawater Pearl Tablet on Body Weight and Behavioral Characteristics of Chronic Sleep Deprivation Mice

Compared to the N group, the final body weight, final open field test score, and final sugar water preference rate of mice in the M and ES groups were found to be significantly decreased (*P <* 0.05); the final body weight and the final open field test score of the mice in the HSPT group were found to be significantly decreased (*P* < 0.05), and there was no significant difference found in the final sugar water preference rate (Tables **2** to **4**).

Compared to the M group, the final body weight, final open field test score, and final sugar water preference rate of mice in the ES and HSPT groups were found to be significantly increased (*P <* 0.05) (Tables **2** to **4**). Compared to the ES group, the HSPT group exhibited no significant difference in the final body weight, final open field test score, and final sugar water preference rate (Tables **2** to **4**).

### 3.2 Effect of Hydrolyzed Seawater Pearl Tablet on the Hippocampal Structure of Chronic Sleep Deprivation Mice

**Fig. (2):**
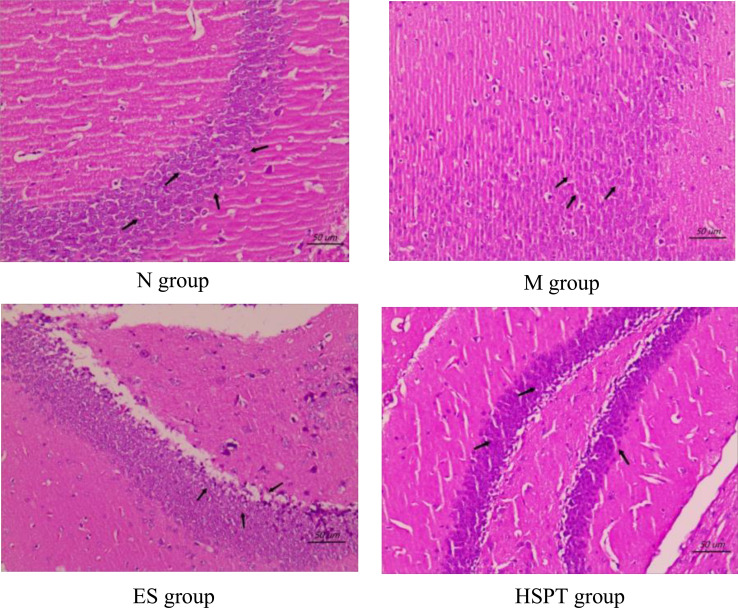
Effect of hydrolyzed seawater pearl tablet on the hippocampal structure of chronic sleep deprivation mice (HE,×400), scale bar = 50μm.

In the N group, the morphology of neurons in the hippocampus was normal, the molecular layer and outer granular layer were well-defined, the boundary was clear, and the pyramidal cell layer was arranged in order (Fig. **2**). In the M group, some neurons in the hippocampus showed pyknosis, deep staining, and cytosolic dissolution. A large number of pyramidal cells became smaller and the distance between cells widened significantly (Fig. **2**). Compared to the M group, the nucleus pyknosis, deep staining, and cytosolic dissolution of neurons in the hippocampus of the ES group and the HSPT group were reduced, the pyramidal cell layers were arranged in order, and the cell spacing basically returned to normal (Fig. **2**).

### 3.3 Effect of Hydrolyzed Seawater Pearl Tablet on Oxidative Stress in the Hippocampus of Chronic Sleep Deprivation Mice

Compared to the N group, the content of MDA in the hippocampus of the M group was significantly increased, and the content of GSH and the activities of SOD and GSH-Px were significantly decreased (*P <* 0.05); the contents of MDA in the hippocampus of the ES and HSPT groups were significantly increased, and the activity of SOD significantly decreased (*P <* 0.05), and there was no significant difference observed in the content of GSH and the activity of GSH-Px (Table **5**).

Compared to the M group, the MDA contents in the hippocampus of ES and HSPT groups were found to be decreased *(P <* 0.05*)* significantly, and the GSH content and SOD and GSH-Px activities were found to be increased (*P <* 0.05) significantly (Table **5**). Compared to the ES group, there was no significant difference found in the contents of MDA, GSH, and the activities of SOD and GSH-Px in the hippocampus of the HSPT group (Table **5**).

### 3.4 Effect of Hydrolyzed Seawater Pearl Tablet on Apoptosis-Related Protein Expression in the Hippocampus of Chronic Sleep Deprivation Mice

**Fig. (3):**
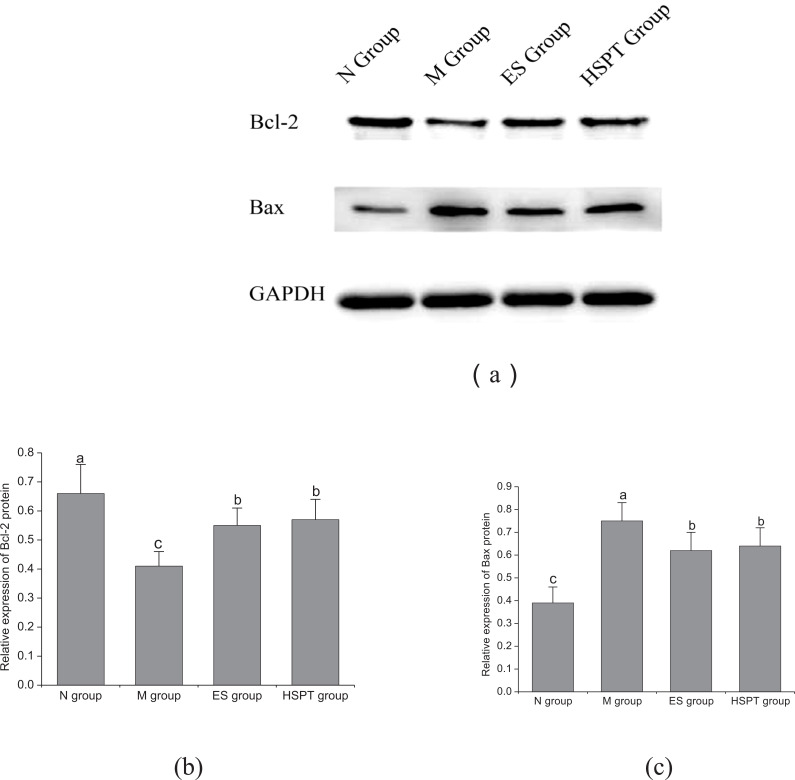
Effect of hydrolyzed seawater pearl tablet on the expression of Bcl-2 (**a**, **b**) and Bax (**a**, **c**) proteins in the hippocampus of chronic sleep deprivation mice; different letters represent significant differences between the two groups.

Compared to the N group, the expressions of Bax protein in the hippocampus of mice in the M, ES and HSPT groups were found to be increased significantly, and the expressions of Bcl-2 protein to be decreased significantly (*P <* 0.05) (Fig. **3**). Compared to the M group, the expressions of Bax protein in the hippocampus of mice in the ES and HSPT groups were found to be significantly decreased (*P <* 0.05), and the expressions of Bcl-2 protein were found to be significantly increased (*P <* 0.05) (Fig. **3**). Compared to the ES group, there was no significant difference found in Bax and Bcl-2 proteins expression in the hippocampus of mice in the HSPT group (Fig. **3**).

### 3.5 Effect of Hydrolyzed Seawater Pearl Tablet on Dna Expression of Five Kinds of Intestinal Bacteria in Chronic Sleep Deprivation Mice

Compared to the N group, the DNA expression of E. coli, Lactobacillus, Bifidobacterium, and Bacteroidetes in the intestine of the M group was found to be significantly decreased (*P <* 0.05), and the DNA expression of *Clostridium perfringens* was found to be significantly increased (*P <* 0.05). The DNA expression of *Clostridium perfringens* in the intestines of mice in the ES group was found to be significantly decreased (*P <* 0.05), and there was no significant difference found in the DNA expression of *E. coli*, Lactobacillus, Bifidobacterium and Bacteroidetes. In the HSPT group, the DNA expression of *E. coli* was found to be increased significantly (*P <* 0.05), while that of Clostridium perfringens decreased significantly in the intestines of mice (*P <* 0.05), and there was no significant difference found among Lactobacillus, Bifidobacterium and Bacteroidetes (Table **6**).

Compared to the M group, the DNA expression of E. coli, Lactobacillus, Bifidobacterium, and Bacteroidetes in the intestine of mice in the ES and HSPT groups was found to be increased significantly (*P <* 0.05), and the DNA expression of Clostridium perfringens to be decreased significantly (*P <* 0.05) (Table **6**). Compared to the ES group, the DNA expression of Bacteroidetes was found to be increased significantly in the HSPT group (*P <* 0.05), and there was no significant difference observed in the DNA expression of *E. coli*, *Lactobacillus*, *Bifidobacterium*, and *Clostridium perfringens* (Table **6**).

## 4 DISCUSSION

Animal behavior evaluation is widely utilized in scientific research, especially in the evaluation of animal models of cognitive dysfunction-related diseases and the study of their physiological mechanisms [16, 17]. This experiment was conducted to investigate the effect of hydrolyzed seawater pearl tablet on chronic sleep deprivation model mice and to record the effect of hydrolyzed seawater pearl tablet intervention on chronic sleep deprivation stress, *i.e*., body weight and behavioral activities of mice. Chronic sleep deprivation was found to significantly decrease the body weight of mice; the result of the open-field experiment showed that chronic sleep deprivation significantly reduced the performance score of mice, indicating that their activity and curiosity about the environment decreased; the result of the sugar water preference experiment showed that chronic sleep deprivation significantly reduced the sugar water preference rate of mice, indicating the lack of pleasure and happiness. Therefore, chronic sleep deprivation induces mice to develop a depression-like mental state, which is consistent with the findings of Ou *et al*. [18]. When 0.16 g/kg hydrolyzed seawater pearl tablet was given to the chronic sleep deprivation mice, the body weight, open-field test score, and sugar preference rate of the mice were found to be significantly increased, and the effect was equivalent to that of estazolam, indicating that 0.16g/kg hydrolyzed seawater pearl tablet can significantly improve the depression-like mental state of the mice caused by chronic sleep deprivation.

Under normal conditions, the body consumes oxygen through mitochondria to produce ROS, which are cleared by antioxidants (such as GSH) and antioxidant enzymes (such as SOD and GSH-Px), so that oxidation and antioxidation mechanisms in the body are in a dynamic balance. When the body is stimulated by the outside world, a large quantity of ROS is produced in the body, which makes the oxidative balance of the body disrupted, leading to DNA damage, lipid peroxidation, tissue damage of the body, and aggravating the occurrence of various diseases [19, 20]. MDA is the product of lipid peroxidation decomposition, and its content is positively correlated with the degree of lipid peroxidation. It is one of the markers reflecting the oxidative damage of the body [21]. The effects of different sleep deprivation durations on oxidative stress of the body are inconsistent. Some studies have shown that the antioxidant defense system of the liver is destroyed, and the activities of SOD and GSH-Px are reduced under the condition of sleep deprivation for 48h, resulting in the failure to clear ROS in time, which leads to oxidative stress tissue damage [22, 23]. This study showed that under the condition of chronic sleep deprivation for 7 days, the content of MDA in the hippocampus of mice increased, the content of GSH and the activities of SOD and GSH-Px decreased, and the tissue structure got damaged. Hydrolyzed seawater pearl tablet significantly reduced the content of MDA in the hippocampus of chronic sleep deprivation mice, increased the content of GSH and the activities of SOD and GSH-Px, and restored the normal tissue structure. The effect was found to be equivalent to that of estazolam. Therefore, the intervention of hydrolyzed seawater pearl tablet can clear excessive ROS in the hippocampus and alleviate tissue structure damage through the antioxidant effect.

Under abnormal conditions, excessive ROS will damage mitochondrial DNA, inhibit the synthesis of ATP by mitochondria, damage cell growth, and accelerate apoptosis. It also causes mitochondria to release cytochrome C to the cytoplasm, activate caspase-3, and induce cell apoptosis [24, 25]. Bcl-2 is an anti-apoptotic protein factor, whereas Bax is an apoptotic protein factor. Bcl-2 protein can inhibit the activation of Bax protein, and the ratio between Bcl-2 and Bax determines whether a cell is apoptotic [26]. It has been found that the phase with the highest ratio of Bcl-2/Bax in the brain of mice is during sleep at night, and after 24h of sleep deprivation, the ratio of Bcl-2/Bax decreases. Supplementary sleep can improve the protein ratio of Bcl-2/Bax in the hippocampus, suggesting that sleep may play a protective role for brain neurons by affecting anti-apoptotic and apoptotic protein factors, such as Bcl-2 and Bax [27]. This study showed that under the condition of chronic sleep deprivation for 7 days, the expression of Bax protein increased, the expression of Bcl-2 protein decreased, and the ratio of Bcl-2/Bax decreased in the hippocampus of mice; hydrolyzed seawater pearl tablet significantly reduced Bax protein expression, increased Bcl-2 protein expression and increased Bcl-2/Bax ratio in chronic sleep deprivation mice, and the effect was found to be equivalent to that of estazolam. Therefore, the intervention of hydrolyzed seawater pearl tablet can alleviate the damage to tissue structure by clearing excessive ROS in the hippocampus, thereby inhibiting apoptosis.

The total genomic information of microbiota is the “intestinal metagenome”, which is second only to the innate genetic genome, as the “second genome” affecting human health and the “super microorganism” of the human body [28]. The DNA qRT-PCR analysis of fecal flora in this study showed the structure and function of microbiota to be dysregulated under the influence of chronic sleep deprivation stress. Clostridium perfringens are recognized as harmful bacteria. When their level in the intestine increased in this study, the balance of microbiota was found to be broken, the barrier protection as weakened, and the invasion of harmful bacteria was found to be increased. Bifidobacterium and Lactobacillus are recognized as beneficial bacteria. Some studies have shown that they can affect the movement and behavior performance of the host. Their reduction in this study further exacerbated the disorder of microbiota [29]. Some studies have used primers designed based on the 16S rRNA gene of E. coli to carry out qRT-PCR to detect the changes in the total amount of microbiota [30], and Bacteroidetes account for a large proportion in the body's intestine [31]. In line with this, the changes in the amount of E. coli and Bacteroidetes in this study also correspond to the changes in the total amount of microbiota, which further indicates that chronic sleep deprivation stress reduces the total amount of microbiota.

This study shows that hydrolyzed seawater pearl tablet can significantly increase the levels of Escherichia coli, Bacteroides, Bifidobacterium, and Lactobacillus in chronic sleep deprivation mice, which are beneficial bacteria in the intestine, and significantly reduce the amount of Clostridium perfringens, which are harmful bacteria in the intestine. The effect is equivalent to that of estazolam, and the effect of promoting the increase of Bacteroides is better than that of estazolam. Therefore, the intervention of hydrolyzed seawater pearl tablet can adjust the microbiota disorder caused by chronic sleep deprivation stress to a certain extent. Intestinal microorganisms are closely related to the brain through the intestine-brain axis. Microbiota not only regulate the function and health of the intestine, but also affect the function of the nervous system, such as the hypothalamus-pituitary-adrenal (HPA) axis [32]. Neurobiological studies have shown that their mutual regulation plays an important role in maintaining gastrointestinal homeostasis [33]. It has been pointed out that depression is accompanied by an enhanced response of the HPA axis to stress, while animal experiments show that the lack of intestinal microorganisms will lead to an enhanced activity of the HPA axis, and the supplement of bifidobacteria can correct the HPA axis [34]. To sum up, the antidepressant-like effect of hydrolyzed seawater pearl tablet may be related to its regulation of microbiota level, which affects the mental and behavioral activities of chronic sleep deprivation mice through the intestine-brain axis.

## CONCLUSION

Hydrolyzed seawater pearl tablet can improve the depression-like mental state of mice caused by chronic sleep deprivation. The mechanism involves improving the antioxidant activity of the hippocampus to eliminate the excessive presence of ROS, which inhibits cell apoptosis and alleviates tissue structure damage. Meanwhile, it may also be involved in adjusting the microbiota level and improving the mental and behavioral activities of chronic sleep deprivation mice through the intestine-brain axis.

## Figures and Tables

**Fig. (1):**
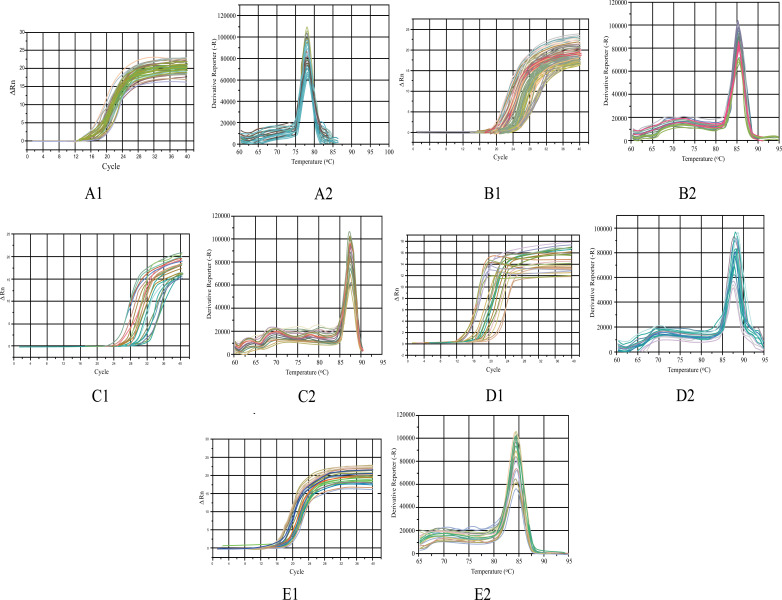
The amplification curve of *E. coli* (**A1**), *Lactobacillus* (**B1**), *Bifidobacterium* (**C1**), *Bacteroidetes* (**D1**), and *Clostridium perfringens* (**E1**); the dissolution curve of *E. coli* (**A2**), *Lactobacillus* (**B2**), *Bifidobacterium* (**C2**), *Bacteroidetes* (**D2**), and *Clostridium perfringens* (**E2**).

**Table 1:** Intestinal bacteria and primers.

**Intestinal Bacteria**	**Amplification Length/bp**	**Forward Primer (5’-3’)**	**Reverse Primer (5’-3’)**
Bacillus bifida	136	GATGCAACGCGAAGAACCTTACCT	CTTAACCCAACATCTCACGACACGA
Bacteroid	132	AAAGGGAGCGTAGGTGGACAGTT	TGCCTTCGCAATCGGAGTTCTTC
Clostridium perfringens	164	GCGTAGAGATTAGGAAGAACACCAG	TATTCATCGTTTACGGCGTGGACTA
Escherichia coli	129	AAACTGGAGGAAGGTGGGGATGA	CCGGACTACGACGCACTTTATGA
Lactobacillus	161	GGGAGGCAGCAGTAGGGAATCTT	GTTAGCCGTGACTTTCTGGTTGGAT

**Table 2:** Effect of hydrolyzed seawater pearl tablet on body weight of chronic sleep deprivation mice.

**Group**	**Initial Body Weight (g)**	**Final Body Weight (g)**
N group	20.52 ± 0.79 ^a^	23.83 ± 1.20^a^
M group	20.09 ± 0.92 ^a^	20.56 ± 0.85^c^
ES group	20.19 ± 0.99 ^a^	21.64 ± 0.78^b^
HSPT group	20.60 ± 1.08 ^a^	21.74 ± 0.67^b^

**Note:** different letters represent significant differences between the two groups.

**Table 3:** Effect of hydrolyzed seawater pearl tablet on open field test score of chronic sleep deprivation mice.

**Group**	**Initial Open Field Test Score**	**Final Open Field Test Score**
N group	87.22 ± 12.73^a^	87.26 ± 11.40^a^
M group	86.52 ± 16.48^a^	34.51 ± 5.27^c^
ES group	87.59 ± 17.03^a^	67.06 ± 8.09^b^
HSPT group	88.90 ± 14.70^a^	70.06 ± 9.32^b^

**Note:** different letters represent significant differences between the two groups.

**Table 4:** Effect of hydrolyzed seawater pearl tablet on sugar water preference rate of chronic sleep deprivation mice.

**Group**	**Initial Sugar Water Preference Rate (%)**	**Final Sugar Water Preference Rate (%)**
N group	75.01 ± 10.73^a^	73.40 ± 7.81^a^
M group	74.02 ± 9.19^a^	47.89 ± 6.16^c^
ES group	73.49 ± 9.01^a^	66.04 ± 7.53^b^
HSPT group	73.97 ± 6.43^a^	69.16 ± 8.44^ab^

**Note:** different letters represent significant differences between the two groups.

**Table 5:** Effect of hydrolyzed seawater pearl tablet on oxidative stress in the hippocampus of chronic sleep deprivation mice.

**Group**	**MDA (nmol/mg)**	**SOD (U·mg)**	**GSH-Px (U·mg)**	**SOD/GSH-Px**	**GSH (μmol/g)**
N group	1.85±0.23^c^	38.24±7.57^a^	15.35±1.36^a^	2.49±0.37^b^	27.87±1.14^a^
M group	6.22±1.31^a^	21.56±6.43^c^	5.27±0.65^b^	4.09±0.46^a^	23.31±2.62^b^
ES group	3.22±0.87^b^	30.15±6.65^b^	13.84±1.97^a^	2.17±0.26^c^	26.26±2.06^a^
HSPT group	3.15±0.79^b^	31.47±5.98^b^	14.57±2.15^a^	2.15±0.29^c^	27.23±1.83^a^

**Note:** (a-c) different letters represent significant differences between the two groups.

**Table 6:** Effect of hydrolyzed seawater pearl tablet on DNA expression of five kinds of intestinal bacteria in chronic sleep deprivation mice.

**Group**	***E. coli***	***Lactobacillus***	**Bifidobacterium**	**Bacteroidetes**	**Clostridium Perfringens**
N group	100.00±31.23^b^	100.00±81.89^a^	100.00±19.56^a^	100.00±27.66^ab^	100.00±16.45^b^
M group	47.78±9.33^c^	27.67±8.65^b^	49.88±16.46^b^	39.79±17.23^c^	196.34±26.22^a^
ES group	124.55±26.74^ab^	83.78±28.89^a^	118.67±36.22^a^	72.36±28.11^b^	47.54±9.17^c^
HSPT group	143.46±31.25^a^	74.56±27.71^a^	127.33±32.56^a^	120.86±28.31^a^	41.45±4.22^c^

**Note:** (a-c) different letters represent significant differences between the two groups.

## Data Availability

The data used to support the findings of this study are included in the article.

## LIST OF ABBREVIATIONS

ES: Estazolam
GSH: Glutathione
GSH-Px: Glutathione Peroxidase
HRP: Peroxidase
HSPT: Hydrolyzed Seawater Pearl Tablet
M: Model Group
MDA: Malondialdehyde
N: Normal Group
ROS: Reactive Oxygen Species
SOD: Superoxide Dismutase
